# Access to treatment in prison: an inventory of medication preparation and distribution approaches

**DOI:** 10.12688/f1000research.23640.3

**Published:** 2020-10-16

**Authors:** Nguyen Toan Tran, Dominique Pralong, Anne-Dominique Secrétan, Audrey Renaud, Gérard Mary, Arnaud Nicholas, Elisabeth Mouton, Clémence Rubio, Célestine Dubost, Francesco Meach, Anne-Claire Bréchet-Bachmann, Hans Wolff

**Affiliations:** 1 Division of Health in Prison, Geneva University Hospitals and the University of Geneva, Chêne-Bourg, Switzerland, 1225, Switzerland; 2 Australian Centre for Public and Population Health Research, Faculty of Health, University of Technology Sydney, Sydney, NSW, 2007, Australia; 3 European Committee for the Prevention of Torture and Inhuman or Degrading Treatment or Punishment (CPT), Council of Europe, Strasbourg, 67075, France

**Keywords:** Access to medication, preparation, dispensing, detention, prison, autonomy, confidentiality, dignity

## Abstract

The preparation and distribution of medication in prisons or jails are critical for individuals to access their treatment. This process is resource-intensive for healthcare professionals and may violate principles of confidentiality, autonomy, respect, and dignity if non-qualified staff are involved. However, there are no published best practices on the topic. This report aims to bridge this gap by presenting the results of a mapping exercise on different models of medication preparation and delivery. Authors call upon healthcare professionals to enrich this live document to inform health services research further and improve access to prescribed medications for people experiencing incarceration.

## Background

Individuals experiencing incarceration carry a high burden of physical and mental health conditions ^1– 4^. Clinical services operating in prisons and jails are vital in offering non-pharmacological and pharmacological interventions to treat, care for, and support incarcerated persons. Once prescribed, medications require coordinated preparation and delivery for individuals to access their treatment on time. The report of the European Committee for the Prevention of Torture and Inhuman or Degrading Treatment or Punishment (CPT) published in 1992 recommended that *there should be appropriate supervision of the pharmacy and the distribution of medicines. Further, the preparation of medicines should always be entrusted to qualified staff (pharmacist/nurse, etc.)* ^5^. Therefore, medication preparation and distribution should only engage qualified healthcare professionals. This process is notably intensive and can take away resources from other clinically meaningful activities, such as individual patient visits and health promotion and prevention activities. In smaller detention facilities (less than 100 occupants), which usually have limited healthcare staff, prison officers or even prisoners can be involved in medication preparation and distribution ^6^. Such practices violate the principles of confidentiality, autonomy, respect, dignity, and quality of care. CPT experts raised such concerns during recent visits in different European countries, where they observed a lack of respect for the 1992 recommendations of the CPT ^7^. For instance, prison officers and incarcerated individuals were found in Greece to work as orderlies (i.e., persons trained in first aid and selected healthcare tasks, such as the delivery of medications, under the supervision of nurses) ^8^. In Norway, although nurses were present daily, custodial officers had the duty to distribute prescribed medications ^9^.

Best practices related to medication preparation and distribution in prison, and in particular in smaller facilities, could help inform the organization of healthcare service delivery that complies with quality of care, confidentiality, and other human rights principles. There is, however, a paucity of publication on the subject. The objective of this paper is to present a live inventory of different approaches to medication preparation and delivery in prisons.

## Methods

First, we looked for published literature on different modalities of medication preparation and distribution. On 15 August 2019, we searched PubMed and Google Scholar for publications studying different approaches using search strings combining medical subject headings (MeSH) terms related to medication preparation, dispensing, and prison with terms related to best practices (i.e., *pharmaceutical preparations AND prisons AND practice guidelines as topic*). The review of titles and abstracts yielded no relevant articles, prompting us to extend our search to the grey literature by using Google Search, to no avail. Though our choice of keywords were limited, the lack of relevant publications yielded by our search suggests there may be paucity of research on this specific yet important operational aspect of health services management in prisons.

Second, we conducted a focus group discussion among our clinical staff from the Division of Health in Prison, which operates at the post-trial detention facility of La Brenaz in Geneva, Switzerland. On 22 August 2019, the Head of the unit facilitated a focus group discussion, which involved four female nurses, two male nurses, two internal medicine specialists (one female, one male), and a female psychiatrist. The discussion was guided by the care continuum of medication preparation, distribution, and self-administration and the “4Ws + H” lens (what, where, when, who, and how). We did not record the discussion but directly captured participants’ inputs on a whiteboard to help visualize the emerging mapping and catalyze additional contributions. Photographs of the whiteboard were taken and used to transcribe and further categorize the information in a Word document table ( Table 1). We consolidated the initial results with inputs from healthcare colleagues who could not attend the focus group discussion and validated the content of the table with participants of the focus group discussion and the Division Chief. The mapping drew from our work experience in prisons and visit to other facilities in Switzerland and various countries in Europe and North America. It was also informed by quality of care and operational considerations with a focus on reducing errors ^10^ and promoting key human rights principles, such as autonomy, confidentiality, respect, and dignity ^11^.

**Table 1.** Summary of different models of medication preparation and delivery in prisons.

Preparation		Comments
**By whom?**	Clinical staff working in prison - nurses or healthcare assistants - doctors - dedicated pharmacy preparer	- in most cases - if no nurses, such as for ambulatory emergencies outside working hours - e.g., in France ( *préparatrice* or *préparateur en* *pharmacie)*
	Prison officers	Raises quality of care and confidentiality issues
**Where?**	Clinics in prison	
	Pharmacies - intra-muros - extra-muros	- e.g., in large detention facilities - e.g., for the preparation of opioid agonist therapy,such as methadone, where this cannot be done in prison. In France, the University Hospital Centers (CHU) have an automated system to prepare medications, which are then delivered to prisons in the form of individual sachets containing de-blistered medicines
	Prison officers’ quarters	Raises quality and confidentiality issues
**How?**	Manually	Time-consuming, prone to errors
	Automated/computerized	Start-up investment required, less time-consuming, less prone to errors
**In which** **form?**	Tablets in the blister pack	Medication quality preserved
	Tablets deblistered and intact	Medication quality can be compromised if not taken immediately or put into an adequate medication container; no blister label to check the expiry date and whether the medication is the correct one.
	Tablets deblistered, crushed, and mixed with water	Medication quality compromised, degrading (no patient autonomy, lack of respect and dignity), prone to wrong medication administration
	Liquid or cream in its original container (e.g., tube or bottle)	Medication quality preserved but often larger quantity than required and may not adhere to prison security requirements (e.g., plastic and transparent containers)
	Liquid or cream in smaller and transparent plastic containers with cover	Medication quality can be compromised if not taken or applied immediately; no original label to check the expiry date and whether the medication is the correct one
Delivery		
**By whom?**	Clinical staff working in prison - nurses or healthcare assistants - doctors	- in most cases - if no nurses (e.g., ambulatory emergencies outside working hours)
	Prison officers	Raises quality, confidentiality and other patients’ rights issues (e.g., coerced medication administration), confusion of roles (prison vs. health staff)
	Fellow incarcerated persons	Raises quality and confidentiality issues
	Educators or teachers	E.g., in facilities for minors; raises quality of care and confidentiality issues
**How?**	In-hand, e.g.,: - in the clinic - at the cell door - at the workplace - in the classroom - at a prison counter	- allows 2-way communication, however access may be limited by restrictive rules regarding movements inside the facility - potential lack of confidentiality - potential lack of confidentiality - potential lack of confidentiality - e.g., reception desk; potential lack of confidentiality
	Left inside the cells	E.g., for individuals living in individual rooms
	Self-service from a locked medication cupboard	E.g., prepared medications are left in individual boxes stored and locked in a common cupboard; prison officers open the cupboard at a set time for patients to take their medications; confidentiality issues, prone to errors, prone to violent interactions between patients
	Personal locked medication boxes	E.g., at the post-trial detention center of La Brenaz, Geneva (168 individual rooms); requires start- up investment; promotion of users autonomy, confidential, dignified, and respectful; no reported theft or peer pressure incidents; possible operational challenges if implemented in larger facilities or pre- trial prisons with high turn-over
Self-administration		
	Under direct supervision - for all prescriptions - for controlled substances	- lack of respect, dignity, and autonomy
	Unsupervised	- confidentiality, autonomy, respect, and dignity preserved; risk of misuse

The Cantonal Ethical Review Board of Geneva granted ethical approval for the study (2017-01379). All participants consented to participate in the study and have the data published.

All the available data is presented in this paper.

## Results

Table 1 summarizes different models of medication preparation and delivery with the right column giving comments on the quality of care and operational considerations as well as human rights principles. Within the same facility, various modalities may coexist, depending on staff availability and medication type. Medication can be prepared manually or via an automated and computerized system by a range of health cadres at different locations, including clinics within the facility, pharmacies inside or outside the facility, or prison officers’ quarters if officers carry such a duty. Medication tablets can be given within blister packs or deblistered (intact or crushed), while liquids or creams remain in their original tubes or bottles or are transferred into plastic containers. The distribution can be the responsibility of clinical staff, prison officers, fellow incarcerated individuals, educators, or teachers. Medication can be given in hand or left inside the cell, a personal locked medication boxes, or a cupboard for self-service. Finally, patients can take their medication under direct supervision or unsupervised.

## Discussion

This report aimed to present an inventory of different medication preparation and delivery models in carceral settings with a focus on whether they respect quality of care and key human rights principles. Ensuring access to medication while conforming to prison security requirements and taking into account concerns about trafficking, theft, and misuse, particularly of prescribed psychoactive substances ^12^, needs a pragmatic and well-adjusted operational approach. We acknowledge the fact that our inventory is not exhaustive – this was the beginning of an effort to bridge the gap in published best practices on the topic. Therefore, we call upon prison health services managers, providers, and researchers to enrich this live document with their own experience and observations by adding their contributions directly in the section entitled “Comments on this article” located at the bottom of the online page of the article (an updated version will be uploaded once information saturation is reached). Additionally, individuals experiencing incarceration should be engaged in programmatic and research discussions to provide their perspectives on the topic so that guidance and practices reflect their needs. This continuously enriched inventory can provide a foundation for further operational research and cost-effectiveness studies. The emerging best practices can help inform the design of new medication delivery systems that can contribute to improve the efficiency of healthcare services in prisons as well as empower individuals to safely, timely, and confidentially access and manage their prescribed treatment.

## Data availability

### Underlying data

All data underlying the results are available as part of the article and no additional source data are required.
