# Supplementary material for: Access to treatment in prison: an inventory of medication preparation and distribution approaches
Source: F1000Res. 2020 Oct 16;9:357. Originally published 2020 May 13. [Version 3] doi: 10.12688/f1000research.23640.3 (PMC7570324; doi:10.12688/f1000research.23640.3)
Supplement: Peer review history

# Peer review: Reviewer response for version 3

**DOI:** 10.5256/f1000research.30223.r73243

**Published:** 2020-10-16

**Authors:** Lamiece Hassan

**Affiliations:** 1 University of Manchester, Manchester, UK

Satisfied with amendments. No further comments.

Is the work clearly and accurately presented and does it cite the current literature?

Partly

If applicable, is the statistical analysis and its interpretation appropriate?

Not applicable

Are all the source data underlying the results available to ensure full reproducibility?

No

Is the study design appropriate and is the work technically sound?

Partly

Are the conclusions drawn adequately supported by the results?

No

Are sufficient details of methods and analysis provided to allow replication by others?

No

Reviewer Expertise:

Prescribing practices in prisons; forensic psychiatric epidemiology; qualitative research.

I confirm that I have read this submission and believe that I have an appropriate level of expertise to confirm that it is of an acceptable scientific standard.

# Peer review: Reviewer response for version 2

**DOI:** 10.5256/f1000research.29916.r72344

**Published:** 2020-10-08

**Authors:** Lamiece Hassan

**Affiliations:** 1 University of Manchester, Manchester, UK

The authors have responded to the points I raised in my initial report. The changes they have made have improved the paper.

 Yet, I still feel that their choice of keywords for the literature review was unduly narrow; thus concluding that there is a paucity of literature after such a limited search may be premature.

 For future work, I would recommend using a broader set of keywords (e.g. pharmacy + prison) to unearth relevant literature, though this will require more time and effort to filter results relevant to their own particular interests. Indeed, having done a brief search using these keywords on PubMed, the first page of results suggested several potentially relevant papers.

 For this reason, may I suggest the following change to the wording:

 'Though our choice of keywords were limited, the lack of relevant publications yielded by our search suggests there may be paucity of research on this specific yet important operational aspect of health services management in prisons.'

Is the work clearly and accurately presented and does it cite the current literature?

Partly

If applicable, is the statistical analysis and its interpretation appropriate?

Not applicable

Are all the source data underlying the results available to ensure full reproducibility?

No

Is the study design appropriate and is the work technically sound?

Partly

Are the conclusions drawn adequately supported by the results?

No

Are sufficient details of methods and analysis provided to allow replication by others?

No

Reviewer Expertise:

Prescribing practices in prisons; forensic psychiatric epidemiology; qualitative research.

I confirm that I have read this submission and believe that I have an appropriate level of expertise to confirm that it is of an acceptable scientific standard, however I have significant reservations, as outlined above.

# Response

**Published:** 2020-10-12

**Authors:** Nguyen Toan Tran

**Affiliations:** University of Geneva, Switzerland

Many thanks for your feedback and advice. We have amended the text accordingly. With much appreciation and warmest regards

# Peer review: Reviewer response for version 1

**DOI:** 10.5256/f1000research.26085.r69115

**Published:** 2020-09-02

**Authors:** Lamiece Hassan

**Affiliations:** 1 University of Manchester, Manchester, UK

This brief report addresses the topic of medication preparation and dispensing in prisons. The authors draw on a focus group discussion and their own previous experiences to map approaches to medication preparation, dispensing and administration, providing a brief synopsis of different models.

 As the authors admit - this approach was not intended to be exhaustive; rather, this contribution marks a preliminary effort to gather more comprehensive evidence of experiences and practices, with the hope of eventually informing best practice. Nonetheless, while I accept and endorse their focus on this important topic, I do have some concerns about the quality of the methods and analysis reported. Attention to these matters would improve the scientific contribution of this paper, even at this early stage.

 First, the authors stated they looked for relevant literature in the published and grey literature and found nothing of relevance. Having authored reports and published papers commenting on pharmacy services and access to medication in prison myself (e.g. medicines reconciliation practices on entry to prison, in possession medication), I find this a bold statement and difficult to reconcile with my own knowledge of the literature. Perhaps it was their choice of keywords, geographical focus, date range or inclusion criteria that explains these limited results, but as currently written I am concerned that they have missed relevant literature commenting on pharmacy services in prison that might be directly or indirectly relevant to framing the topic.

 Second, even for a brief scientific report there is very little information about the focus group discussion. There are limited details about the conduct and approach to analysis that would normally be required to satisfy notions of replicability (e.g. Was a topic guide used? Who moderated the group? Was it recorded, transcribed, coded and/or otherwise thematically analysed?). No reference is provided for the mapping approach mentioned and no direct quotations are provided so it is difficult to assess the quality of this approach as an empirical method for data collection and analysis.

 Third, despite my reservations about how it was produced, I do think the table summarising different models of medication preparation and dispensing could potentially be a useful direction to pursue. However, as it is currently presented in a static format, it is not clear how this will become a ‘live document’ (as the authors envisage) that others can actively contribute towards in future. If the paper is published here, this presents an important opportunity to publicise their work and enable others to contribute examples (perhaps of literature and practice). Will/can the authors capitalise on this by including a mechanism for submitting examples e.g. a link to a live document that others can actively add to (e.g. Google docs or a survey)?

Is the work clearly and accurately presented and does it cite the current literature?

Partly

If applicable, is the statistical analysis and its interpretation appropriate?

Not applicable

Are all the source data underlying the results available to ensure full reproducibility?

No

Is the study design appropriate and is the work technically sound?

Partly

Are the conclusions drawn adequately supported by the results?

No

Are sufficient details of methods and analysis provided to allow replication by others?

No

Reviewer Expertise:

Prescribing practices in prisons; forensic psychiatric epidemiology; qualitative research.

I confirm that I have read this submission and believe that I have an appropriate level of expertise to confirm that it is of an acceptable scientific standard, however I have significant reservations, as outlined above.

# Response

**Published:** 2020-09-25

**Authors:** Nguyen Toan Tran

**Affiliations:** University of Geneva, Switzerland

Dear Reviewer,

We are grateful for your review and have addressed the three important points you raised as follows.

With sincere appreciation

*****

**First, on the literature search:**

We were also surprised by the findings of our search on 15 August 2019. On 25 September 2020, we ran again the search on PubMed using the same combination of terms as described in our report—again, without any limitations (date, language, geography, etc.):

(prisons[MeSH Terms]) AND ("Practice Guidelines as Topic"[Mesh]) AND ("pharmaceutical preparations"[MeSH Terms] OR pharmaceutical preparations[Text Word])

There was 1 result, but of no relevance: Brown L, Takeuchi D, Challoner K. Corneal abrasions associated with pepper spray exposure. Am J Emerg Med. 2000 May;18(3):271-2. doi: 10.1016/s0735-6757(00)90120-7. PMID: 10830682.

We agree that our choice of terms and combinations may have explained the limited results. As our work on this topic moves forward, we will make sure to look into alternative search strategies.

**Second, on the qualitative component: **We have now described this step more explicitly in the manuscript. It now reads as follows:

*Second, we conducted a focus group discussion among our clinical staff from the Division of Health in Prison, which operates at the post-trial detention facility of La Brenaz in Geneva, Switzerland. On 22 August 2019, the Head of the unit facilitated a focus group discussion, which involved four female nurses, two male nurses, two internal medicine specialists (one female, one male), and a female psychiatrist., resulted in a preliminary mapping. We captured the mapping onto a whiteboard to facilitate participants’ inputs before categorizing the The discussion was guided by the care continuum of medication preparation, distribution, and self-administration and the “4Ws + H” lens (what, where, when, who, and how). We did not record the discussion but directly captured participants’ inputs on a whiteboard to help visualize the emerging mapping and catalyze additional contributions. Photographs of the whiteboard were taken and used to transcribe and further categorize the information in a Word document table ( Table 1). We consolidated the initial results with inputs from healthcare colleagues who could not attend the focus group discussion and validated the content of the table with participants of the focus group discussion and the Division Chief. The mapping drew from our work experience in prisons and visit to other facilities in Switzerland and various countries in Europe and North America. It was also informed by quality of care and operational considerations with a focus on reducing errors ^10^and promoting key human rights principles, such as autonomy, confidentiality, respect, and dignity ^11^.*

**Third, on the table:**

We opted for F1000Research as it allowed two features to update our table, which we have now added to the manuscript:

- First, the comment section enables readers to enrich the table
- Second, the paper can be resubmitted with an updated version [Update]: “ *[Update] is a new version, often after the article is indexed and/or the peer review is considered complete, in which authors can add small developments relevant to the research discussed in that article*” – see https://f1000research.com/for-authors/article-guidelines-new-versions

Our addition reads as follows:

*Therefore, we call upon prison health services managers, providers, and researchers to enrich this live document with their own experience and observations by adding their contributions directly in the section entitled “Comments on this article” located at the bottom of the online page of the article (an updated version will be uploaded once information saturation is reached).*

# Peer review: Reviewer response for version 1

**DOI:** 10.5256/f1000research.26085.r63450

**Published:** 2020-06-16

**Authors:** Saman Zamani

**Affiliations:** 1 The Global Fund to Fight AIDS, Tuberculosis and Malaria, Geneva, Switzerland

The brief report titled "Access to treatment in prison: an inventory of medication preparation and dispensing approaches" is an important note for further dialogue among public health experts in this field. 

 The study team appropriately started their inquiry with the health care providers and analyzed/documented important qualitative insight from the initial respondents.

 It is important that the qualitative interviews and discussion to continue engaging the beneficiaries, inmates so to understand their perspectives and finding a practical arrangement for proper health service provision while ensuring respect to their rights. I would suggest this great and primary interviews to be continued among inmates and non-health staff of the prison. 

 Great work and thanks for all efforts and sharing the insight.

Is the work clearly and accurately presented and does it cite the current literature?

Yes

If applicable, is the statistical analysis and its interpretation appropriate?

Not applicable

Are all the source data underlying the results available to ensure full reproducibility?

Yes

Is the study design appropriate and is the work technically sound?

Yes

Are the conclusions drawn adequately supported by the results?

Yes

Are sufficient details of methods and analysis provided to allow replication by others?

Yes

Reviewer Expertise:

Public Health specialist

I confirm that I have read this submission and believe that I have an appropriate level of expertise to confirm that it is of an acceptable scientific standard.

# Response

**Published:** 2020-09-25

**Authors:** Nguyen Toan Tran

**Affiliations:** University of Geneva, Switzerland

Dear Reviewer,

We are grateful for your review and for your suggestion regarding the continuous engagement of people experiencing incarceration, which we have now added to our manuscript. 

With sincere appreciation
